# Supplementary material for: A single nanobody neutralizes multiple epochally evolving human noroviruses by modulating capsid plasticity
Source: Nat Commun. 2023 Oct 16;14:6516. doi: 10.1038/s41467-023-42146-0 (PMC10579229; doi:10.1038/s41467-023-42146-0)
Supplement: Supplementary file 1 — Supplementary Information [file 41467_2023_42146_MOESM1_ESM.pdf]

Supplementary Information

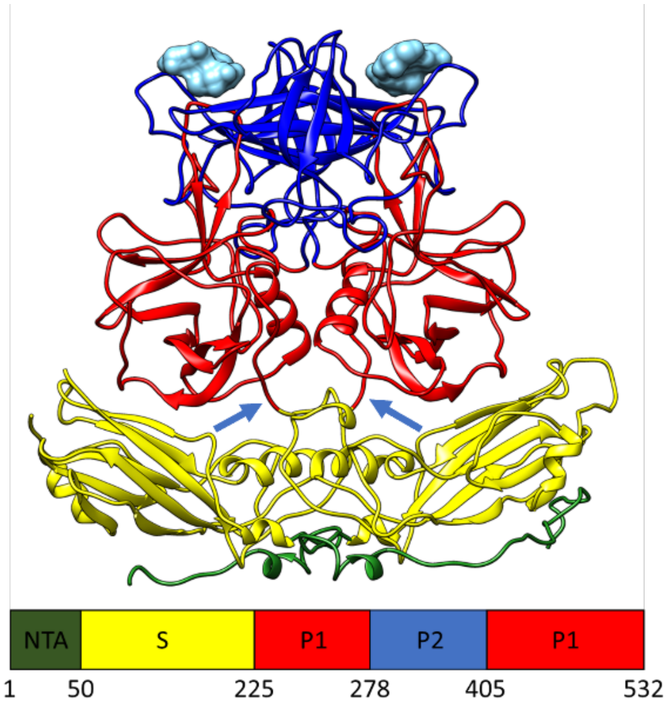

Supplementary Figure 1. Domain organization in GII.4 VP1 and HBGA binding location. A ribbon representation of the structure of the GII.4 capsid protein, VP1, dimer (PDB 7K6V). The NH2-terminal arm (NTA) (residues 10 to 49), which faces the interior of the capsid, is shown in green; the S domain (residues 50 to 225), in yellow; the P1 subdomain (residues 226 to 278, and residues 406 to 520), in red; and the P2 subdomain (residues 279 to 405), in blue. The hinge region between the S- and the P-domain is indicated by an arrow. The surface representation of HBGA ligand is shown in cyan based on the crystal structure HBGA bound complex (PDB 5J35). Structure visualization was prepared using Chimera 1.17.3

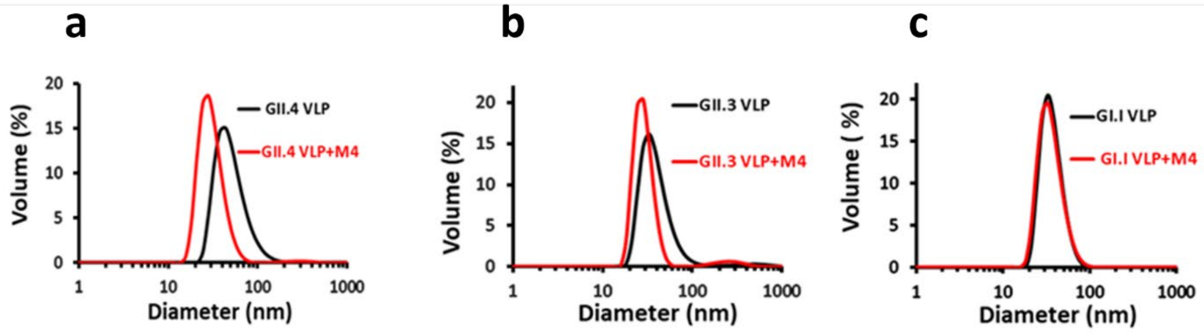

Supplementary Figure 2. Dynamic light scattering (DLS) of GII.3, GII.4, and GI.1 VLPs treated with M4 nanobody. DLS of **a** GII.3 VLP, **b** GII.4 VLP, **c** GI.1 VLP with and without M4 nanobody treatment at a 1 to 4 molar ratio. In all the graphs, the black line represents VLPs alone, and the red line represents VLPs treated with M4 nanobody. Dynamic light scattering profiles were generated using Zetasizer Software 7.13.

a

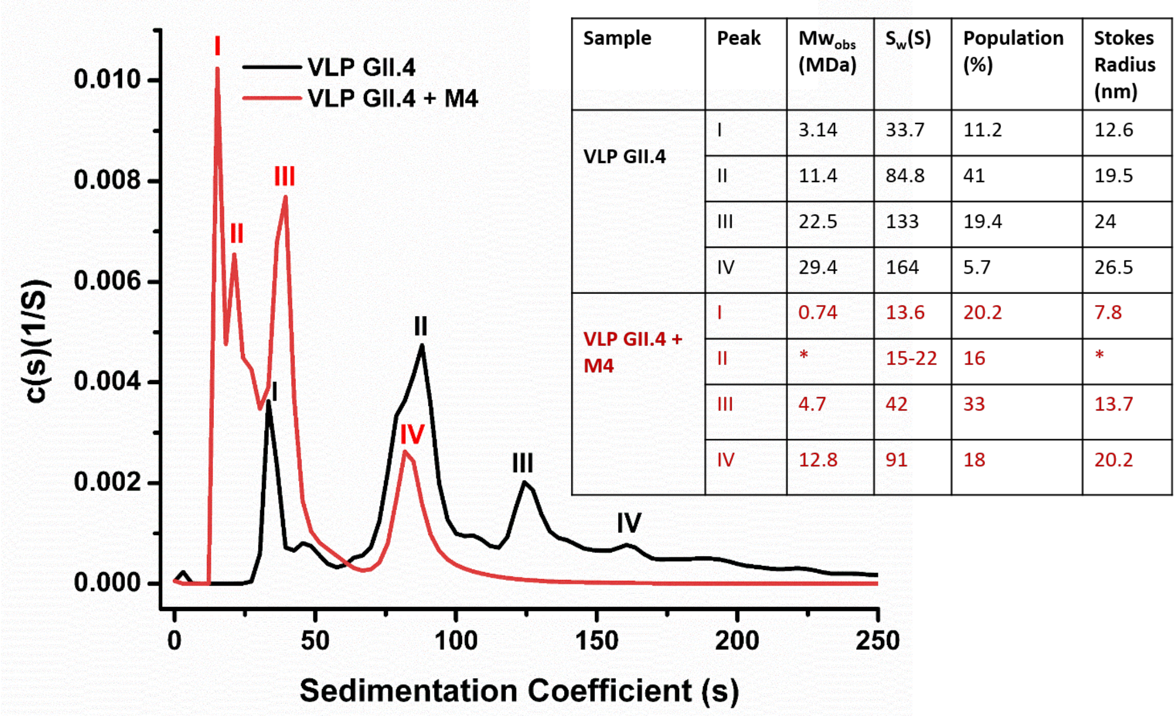

Sample	Peak	Mw_{obs} (MDa)	S_w(S)	Population (%)	Stokes Radius (nm)
VLP GII.4	I	3.14	33.7	11.2	12.6
	II	11.4	84.8	41	19.5
	III	22.5	133	19.4	24
	IV	29.4	164	5.7	26.5
VLP GII.4 + M4	I	0.74	13.6	20.2	7.8
	II	*	15-22	16	*
	III	4.7	42	33	13.7
	IV	12.8	91	18	20.2

b

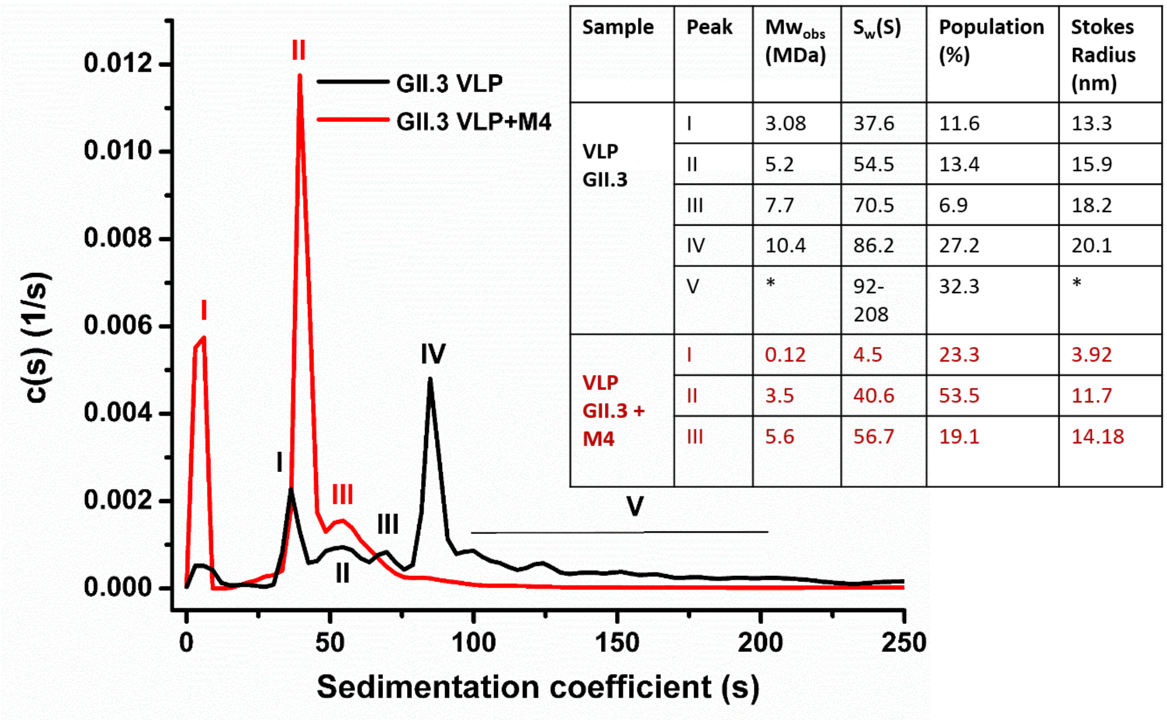

Sample	Peak	Mw_{obs} (MDa)	S_w(S)	Population (%)	Stokes Radius (nm)
VLP GII.3	I	3.08	37.6	11.6	13.3
	II	5.2	54.5	13.4	15.9
	III	7.7	70.5	6.9	18.2
	IV	10.4	86.2	27.2	20.1
	V	*	92-208	32.3	*
VLP GII.3 + M4	I	0.12	4.5	23.3	3.92
	II	3.5	40.6	53.5	11.7
	III	5.6	56.7	19.1	14.18

c

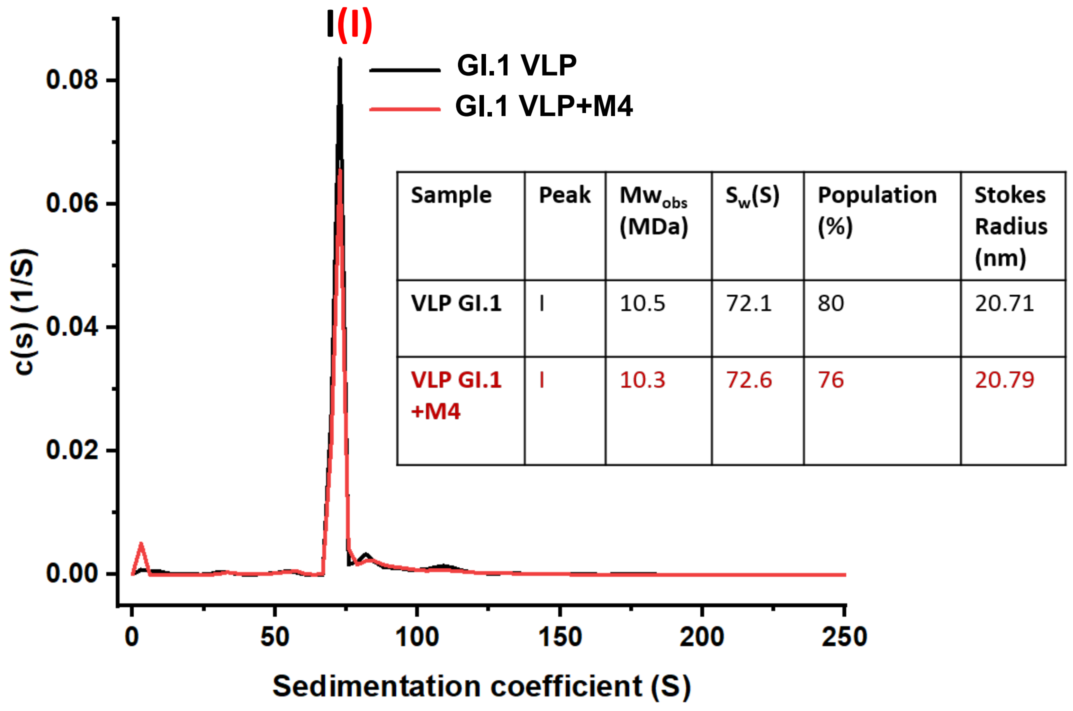

Sample	Peak	Mw_{obs} (MDa)	S_w(S)	Population (%)	Stokes Radius (nm)
**VLP GI.1**	I	10.5	72.1	80	20.71
**VLP GI.1 +M4**	I	10.3	72.6	76	20.79

Supplementary Figure 3. Sedimentation velocity-analytical ultracentrifugation (SV-AUC) of GII.4, GII.3, and GI.1 VLPs treated with M4 nanobody. SV-AUC analysis of **a** GII.4 VLP, **b** GII.3 VLP, and **c** GI.1 VLP with and without M4 nanobody treatment at a 1 to 4 molar ratio. In all the graphs, the black line and peak numbers represent VLPs alone, and the red line and peak numbers represent VLPs treated with M4 nanobody. A table summarizing the different parameters obtained from SV-AUC analysis is also provided with each graph. SV-AUC analysis indicates the disassembly of M4-treated GII.3 and GII.4 VLPs, in contrast to M4-treated GI.1 VLPs used as a negative control. *For shoulder and minor peaks, the software does not compute the hydrodynamic values, these peaks, however, were integrated to obtain the Sw range and % population.

Biotinylated GII.4 P-domain Against M4 Nanobody					
KD(M)	KD error	K_{on}(1/Ms)	K_{dis}(1/s)	Full X^2	Full R^2
<1.0 x 10^{-12}	7.17x 10^{-12}	1.25 x 10^{6}	<1.0 x 10^{-7}	0.06097	0.9936

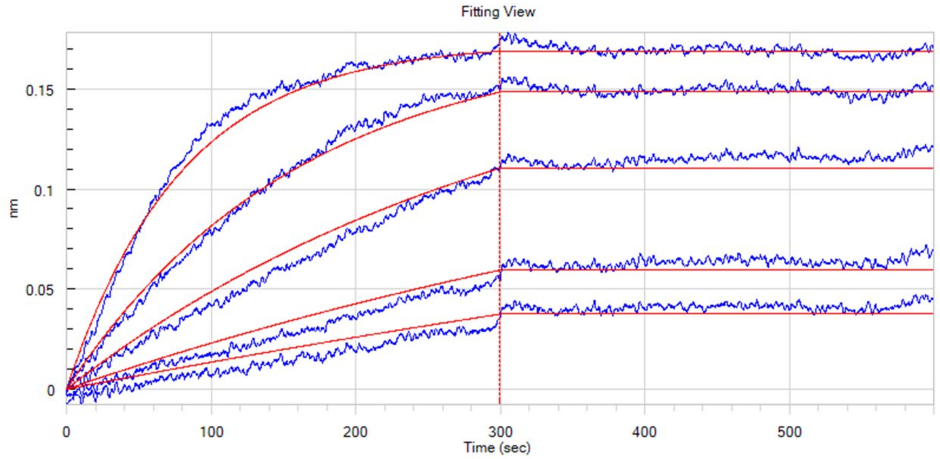

Biotinylated GII.3 P-domain Against M4 Nanobody					
KD(M)	KD error	K_{on}(1/Ms)	K_{dis}(1/s)	Full X^2	Full R^2
4.80 x 10^{-10}	7.36 x 10^{-12}	3.51 x 10^{6}	1.68 x 10^{-3}	0.05243	0.9684

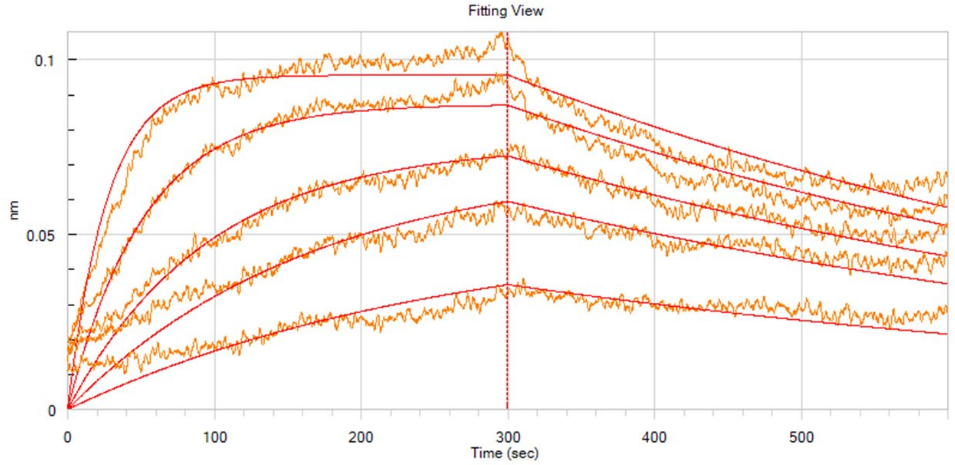

Supplementary Figure 4. Biolayer Interferometry analysis of M4. The binding profiles for biotinylated GII.4 (blue) and GII.3 (orange) P-domains against M4 nanobody. Red lines correspond to fitting curves based on 1 to 1 binding model. Data were analyzed using ForteBio Data Analysis Software Version 7.1.0.38 to determine binding values.

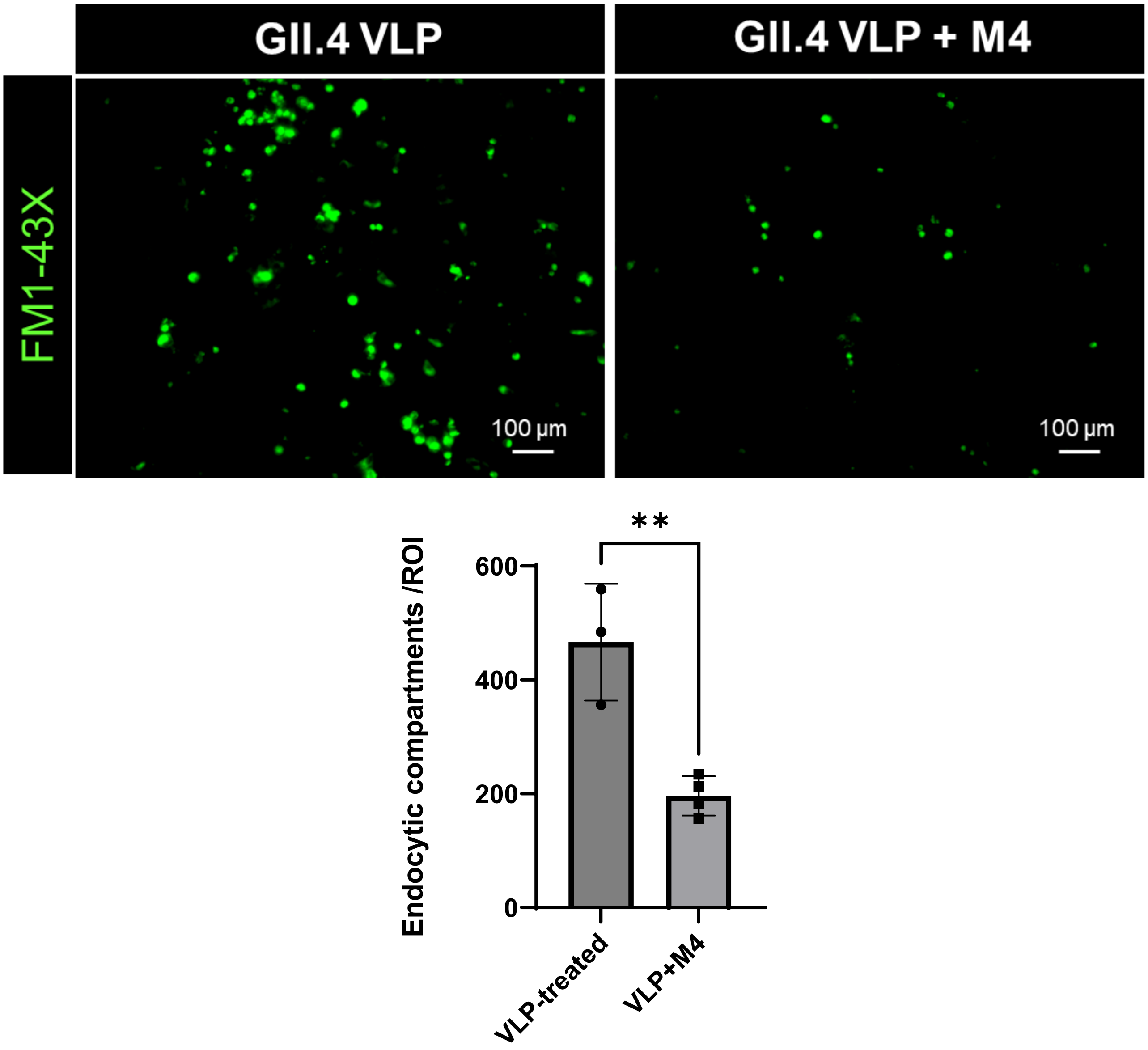

Supplementary Figure 5. Endocytosis analysis of GII.4 VLPs in the presence of M4. Endocytosis was evaluated using FM1-43X dye (green) in the presence of GII.4 Sydney VLPs. FM1-43X uptake indicates GII.4 VLP-induced endocytosis with or without M4 (n=3 HIE replicates). Top Panels: representative images of HIEs with GII.4 VLPs alone or in the presence of M4. Lower panel: FM1-43X positive compartments in HIEs incubated with VLPs with or without M4.
